# A novel *de novo* microdeletion at 17q11.2 adjacent to *NF1* gene associated with developmental delay, short stature, microcephaly and dysmorphic features

**DOI:** 10.1186/s13039-016-0251-y

**Published:** 2016-05-31

**Authors:** Bobo Xie, Xin Fan, Yaqin Lei, Rongyu Chen, Jin Wang, Chunyun Fu, Shang Yi, Jingsi Luo, Shujie Zhang, Qi Yang, Shaoke Chen, Yiping Shen

**Affiliations:** Department of Genetic and Metabolic Central Laboratory, Guangxi Maternal and Child Health Hospital, No. 59, Xiangzhu Road, Nanning, China; Department of Laboratory Medicine, Boston Children’s Hospital, 300 Longwood Avenue, Boston, MA 02115 USA

**Keywords:** Developmental delay, Short stature, Microcephaly, Chromosomal microarray, SNP array, 17q11.2, Microdeletion

## Abstract

**Background:**

Microdeletions at 17q11.2 often encompass *NF1* gene, is the cause for NF1 microdeletion syndrome. Microdeletion at 17q11.2 without the involvement of *NF1* gene is rarely reported.

**Case presentation:**

Here we reported a patient carrying a novel *de novo* deletion at 17q11.2 adjacent to *NF1* gene, who presented with developmental delay, short stature, postnatal microcephaly, underweight and dysmorphic features including flat facial profile, dolicocephaly, hypertelorism, short philtrum, flat nasal bridge and posteriorly rotated and low set ears. Chromosomal microarray analysis revealed a 1.69 Mb *de novo* deletion at 17q11.2 adjacent to *NF1* gene, which involves 43 RefSeq genes. We compared this with four overlapping deletions at this interval.

**Conclusions:**

A rare *de novo* microdeletion at 17q11.2 not involving *NF1* gene is associated with developmental delay and dysmorphic features. Seven genes, *TAOK1*, *PHF12*, *NUFIP2*, *SLC26A4*, *SEZ6*, *GIT1* and *TRAF4* are possible candidates for the clinical features of our patient. The delineation of this rare deletion and description of associated clinical phenotypes will help to understand the genotype-phenotype correlation of genomic imbalances at this locus.

## Background

Chromosomal microarray analysis (CMA) has been extensively used to investigate submicroscopic copy number variants (CNVs) that were not detectable by karyotyping [1]. It has been applied to examining patients with developmental delay/intellectual disability, autism spectrum disorders, and multiple congenital anomalies [2, 3].

Microdeletions at 17q11.2 region often involve a heterozygous 1.5 Mb deletion including *NF1* gene, known as NF1 microdeletion syndrome, which is responsible for 5–20 % of all patients with neurofibromatosis type 1 (NF1) [4]. The deletion was mediated by non-allelic homologous recombination between the *NF1* repetitive sequence (REP) [5]. Patients with the typical 1.5 Mb deletion are also known to have more severe phenotype than NF1 patients caused by point mutations, which mean that more than one gene in the 17q11.2 region may be involved with clinical phenotypes [6]. Microdeletion at 17q11.2 without the involvement of *NF1* gene has not been reported. Here, we described a novel microdeletion at 17q11.2 adjacent to *NF1* gene in a patient with developmental delay and dysmorphic facial features.

## Case presentation

The patient was born to a healthy unrelated 25-year-old mother and a 27-year-old father without family history of multiple congenital anomalies, intellectual disability, recurrent pregnancy loss, or infertility. The girl was born by natural delivery at 37 weeks of gestation. Her birth weight was 2.520 kg (approximately, -2SD), length 48 cm (approximately, -1SD), and head circumference 33.5 cm (approximately, -1SD). At birth, her metabolic and neonatal hearing screening were all normal.

**Fig. 1:**
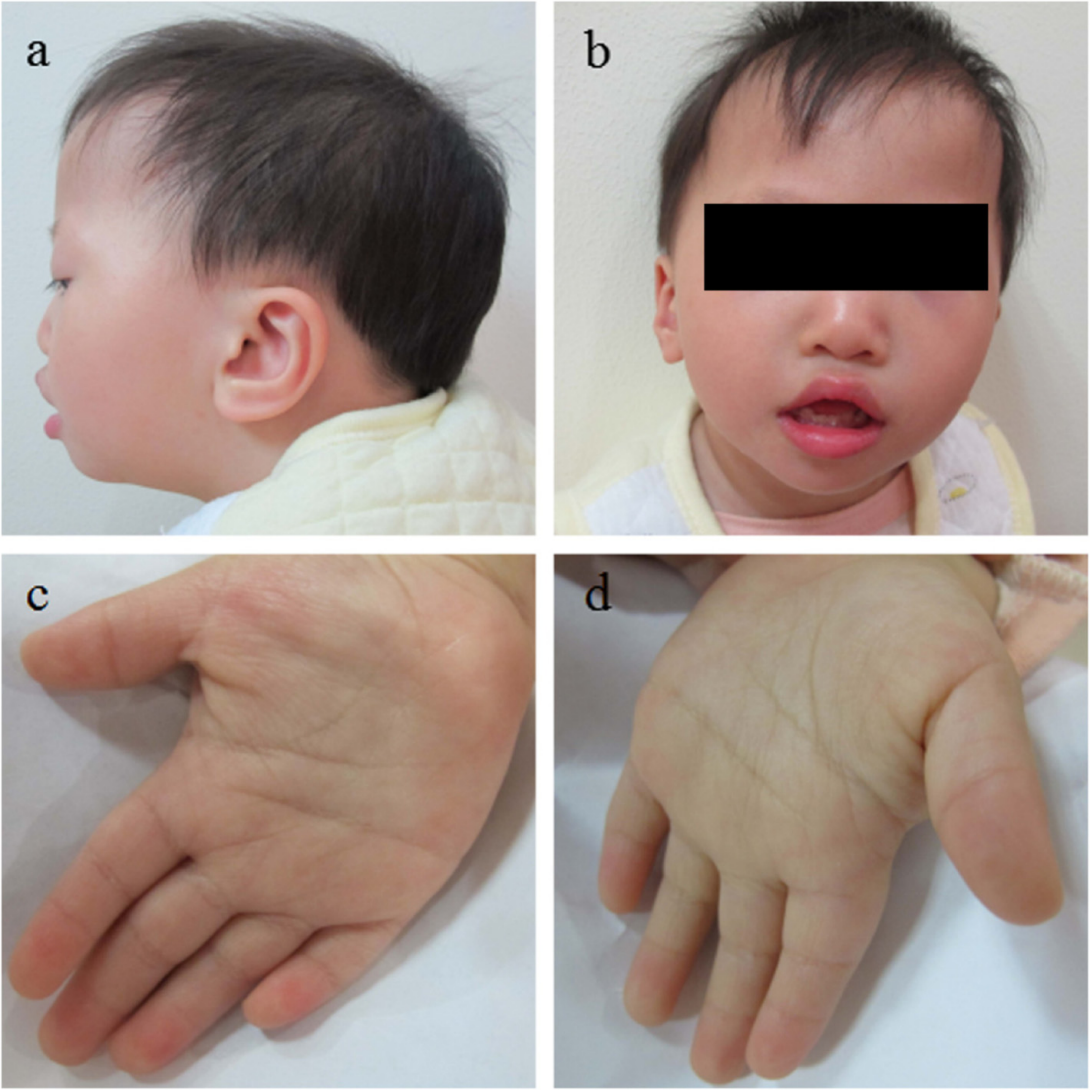
Clinical features of the patients. Note the facial profile, dolicocephaly and low-set posteriorly rotated ear (**a**); hypertelorism, low nasal bridge and short philtrum (**b**); short fifth fingers (**c** and **d**)

Her develop milestones were delayed. Head holding was acquired at 6 months of age, she started to sit up at 10 months and walked with support at 21 months. At age of 21 months, her weight was 7.6 kg (approximately, -3SD), her height was 76 cm (approximately, -2SD), and her head circumference was 43.2 cm (approximately, -3SD). She presented with mild dysmorphic features including flat facial profile, dolicocephaly, hypertelorism, short philtrum, flat nasal bridge and posteriorly rotated and low set ears. She also had a short fifth finger on both hand (Fig. 1). Developmental Screening Test (DST) showed that the DQ and MI scores was 58 and 55, respectively.

## Methods and results

DNA samples were extracted from peripheral blood of the trios using Lab-Aid DNA kit (Zeesan Biotech Co, Ltd, China), DNA concentration was determined with NanoDrop ND-2000 spectrophotometer and soft-ware (NanoDrop Technologies, Berlin, Germany). Genomic wide single nucleotide polymorphism (SNP) array analysis was performed using the Illumina infinium-cytosnp-850 k, which includes over 850 k SNPs in the human genome. Hybridization and array scanning were performed according to the manufacturer’s instruction. Data were analyzed with Illumina Genome Studio and KaryoStudio software. CNVs identified in the samples were visualized by using the UCSC Genome Browser website (http://genome.ucsc.edu) and compared to the Database of Genomic Variants (http://projects.tcag.ca/variation) to exclude CNVs considered as benign variants. The Decipher database and CNV Morbidity Map of Developmental Delay were consulted as resources to aid genotype-phenotype correlation.

**Fig. 2:**
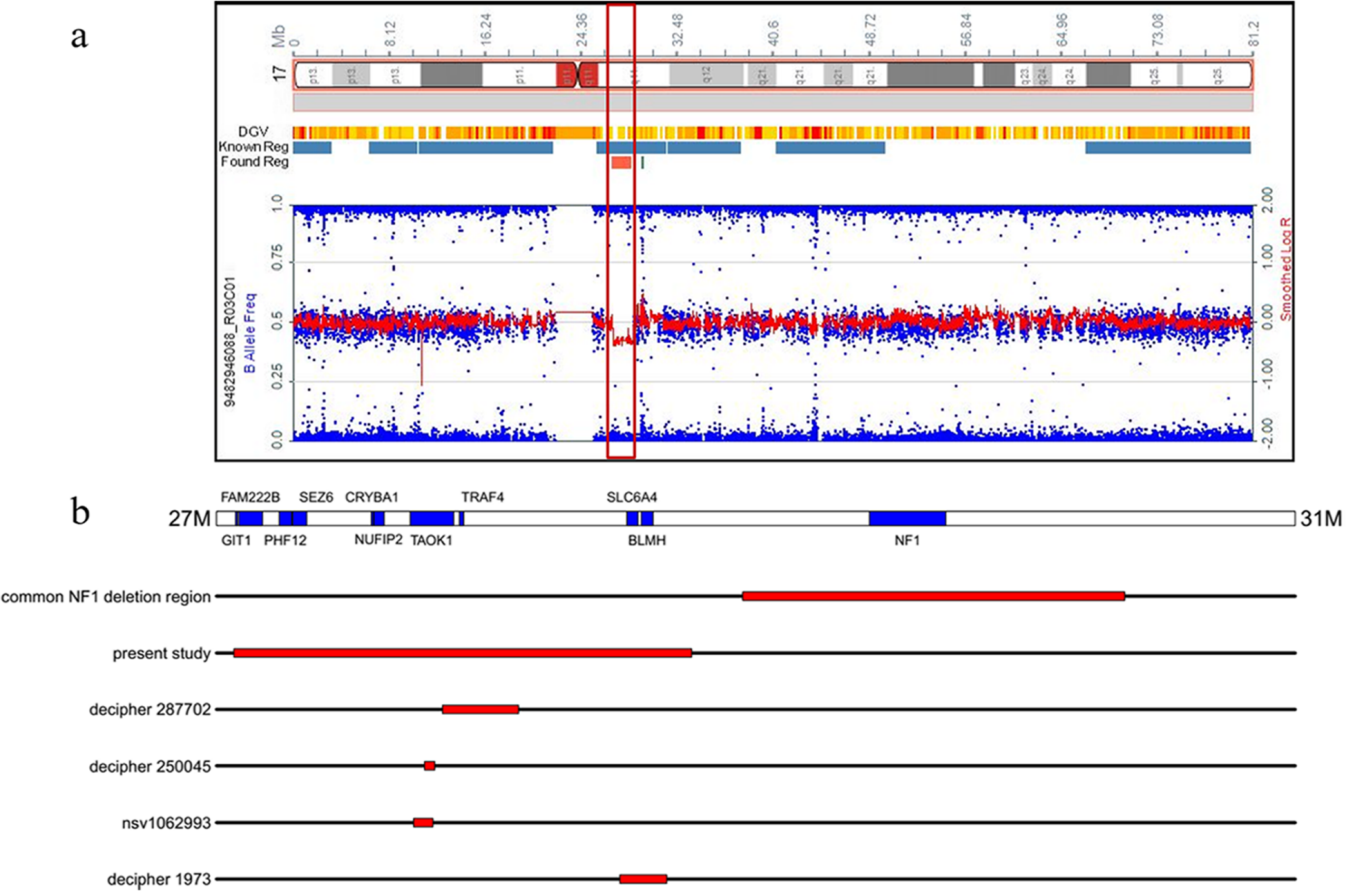
SNP array profile showing a deletion on 17q11.2 (**a**), and schematic representation of 27 Mbp to 31Mbp in 17q11.2 region (**b**). Blue bars represent genes discussed in this paper. Also shown, the extent of the deletions (in red) observed in reported cases are compared with the presented case

A deletion of 1,697,561 bp on chromosome 17 (Fig. 2a) was detected (chr17:27,064,286-28,761,847) (hg19). This position correspond to cytogenetic bands 17q11.2. The chromosomal constitution of the patient was reported as following: arr17q11.2(27064286-28761847) × 1. The parents did not carry the 1.69 Mb copy number variant (CNV), indicating a *de novo* origin of the rearrangement. The deletion involves 43 genes.

## Discussion

Here we presented a 17q11.2 *de novo* deletion characterized by SNP array in a patient with developmental delay and mild dysmorphic features. This is a novel deletion, relative large in size and not listed among the reported CNVs in phenotypically normal individuals in the Database of Genomic Variants, therefore, it is considered as a likely pathogenic CNV.

We searched literature and databases for overlapping deletions at this interval without the involvement of *NF1* gene. There are only four deletion cases currently reported within this interval, all are much smaller in size (Fig. 2b). One (nsv1062993) is reported in the morbidity map of developmental delay, and the other three (#250045, #287702, #1973) are reported in the Decipher database. The case #250045, presented with microcephaly and seizures, carrying a *de novo* deletion of 38 kb (chr17:27771342-27809321) involving part of *TAOK1* gene. The case with 72 kb deletion (chr17:27730573-27802767) from the morbidity map of developmental delay (nsv1062993) also intercept with *TAOK1* gene but the inheritance status and detailed clinical phenotypes are not known, except the patient is presumed to have developmental delay. The deletion nsv1062993 completely overlaps the CNV of #250045, and the two partial overlap *TAOK1* gene, we assume that *TAOK1* gene plays an important role in the phenotype of patient. The patient #287702 carried a *de novo* deletion of 17q11.2 (chr17:27837697-28120076) and a maternally inherited duplication of 15q13.3, and showed dysphasia, poor motor coordination and specific learning disability. The patient #1973 carried a deletion of 173 kb (chr17:28496019-28669139) in size, involving genes including *SLC6A4, BLMH and part of NSRP1*. He showed depression, intellectual disability and psychosis. The breakpoints of this four cases are different, however, their phenotype have some common ground, mainly defined on the basis of the central nervous system.

There are a total of 43 RefSeq genes involved in the deletion in our patient. Seven genes (*FAM222B*, *BLMH*, *PHF12*, *CRYBA1*, *NUFIP2*, *TAOK1* and *SLC6A4*) were predicted to have a haploinsufficiency score less than 10, suggestive of possible clinical consequences with one copy deletion. Little clue is available about the potential relationship of *FAM222B* and *BLMH* with neurodevelopment. *PHF12* is a type of PHD (plant homeodomain) finger protein, acts as a transcriptional repressor. Several proteins with PHD finger are known to epigenetically regulate gene expression and loss of function mutations are associated with intellectual disability phenotypes in human [7, 8]. *CRYBA1* is one of the β-crystallins families, encoding both the beta-A3- and beta-A1-crystallins. Different beta-crystallin proteins can interact with each other and other lens proteins, which play a key role in maintaining the transparency of the lens. The mutations of *CRYBA1* may be destroy the structure and function of crystallins leading to abnormalities in the development and maturation of the retinal vasculature, and have been identified to be causative for congenital cataracts [9, 10]. Up to now, the patient did not present typical phenotype of congenital cataracts, it may be associated with the phenotypic heterogeneity of congenital cataracts.

*NUFIP2* (nuclear fragile X mental retardation protein interacting protein 2) gene encodes an 82-kD protein (called 82-FIP), distributed in various regions of the brain. It was demonstrated that NUFIP2 interacts with FMRP, whose absence causes the fragile-X syndrome [11]. 82-FIP might have a role in the development of the nervous system and in cognitive function. The deregulation of NUFIP2 was reported to be associated with mental retardation or cognitive impairment [12].

The gene of *TAOK1* has the lowest predicted haploinsufficiency score. It encodes hTAOK1, which is a member of the Ste20 group of kinases with the kinase domain located at the N-terminus. hTAOK1 was initially cloned from human fetal brain [13], and highly expressed in human brain, as shown by Northern analysis (http://www.kazusa.or.jp/huge/gfpage/KIAA1361/). TAOK1 may play a role in the developing human brain by inducing neuronal apoptosis and regulating microtubule dynamics and checkpoint signaling [14, 15]. Of significance, the case nsv1062993 and #250045 both partially overlapped *TAOK1* gene, presenting developmental delay and microcephaly, respectively. Accordingly, we hypothesize that *TAOK1* might be involved in the developmental delay and microcephaly in our patient. In addition, a closely related family member TAOK2 is regarded as a autism spectrum disorder susceptibility gene, are shown to regulated basal dendrite development in cortical neurons [16].

The solute carrier family 6 (serotonin neurotransmitter transporter) member 4 gene (*SLC6A4*) encodes an integral membrane protein that transports the neurotransmitter serotonin from synaptic spaces into presynaptic neurons. Therefore, *SLC6A4* gene may play a role in terminating the synaptic actions of serotonin and recycles it into the neurotransmitter pool [17]. Allelic heterogeneity at this gene have been implicated in speech delay, atypical autism, anxiety and obsessive compulsive disorder [18].

In addition, several other genes are known to be functionally important for the development and function of the central nervous system.

SEZ6 (seizure related 6 homolog (mouse)) specific expresses in the brain, especially in the developing forebrain [19]. SEZ6 may have the function on cell adhesion or recognition and protein-protein interaction [20]. The mutations of *SEZ6* were associated with febrile seizures and epilepsy [21].

GIT1 (G protein-coupled receptor kinase interacting ArfGAP 1) is a multifunctional signaling adaptor protein. GIT1 interacts with various proteins and forms signaling complex to modulate the development of dendritic spines and neuronal synapses [22]. Git1^–/–^ mice and dGit^ex21C^ Drosophila mutant were studied and displayed a microcephaly-like brain size reduction decreased neuronal cell body size, and behavioral deficits such as impaired motor coordination and learning [23].

*TRAF4* gene encodes a member of the TNF receptor associated factor (TRAF) family. TRAF proteins are associated with, and mediate signal transduction from members of the TNF receptor superfamily. TRAF4 protein has been shown to interact with neurotrophin receptor, p75 (NTR/NTSR1), and negatively regulate NTR induced cell death and NF-kappa B activation [24]. TRAF4-deficient mice exhibited a high incidence of spina bifida, a defect likened to neural tube defects (NTDs), which revealed that TRAF4 participates in neurulation in vivo [25].

Thus, multiple genes at this interval are likely contributing to the clinical presentations of our patient. Further study is warranted to understand the underlying pathological mechanism.

## Conclusions

We described a patient with developmental delay, short stature, postnatal microcephaly, underweight and dysmorphic features. A novel deletion adjacent to the *NF1* locus was detected. Several genes are functionally important for neurodevelopment and candidate for this novel microdeletion disorder. Additional overlapping cases will help to better understand the clinical presentation of this disorder and critical genes involved.

## Abbreviations

CMA, chromosomal microarray analysis; CNV, copy number variant; DQ, developmental quotient; MI, mental index; RefSeq, reference sequence; SNP, single nucleotide polymorphism
